# Compound Stimulus Presentation Does Not Deepen Extinction in Human Causal Learning

**DOI:** 10.3389/fpsyg.2017.00120

**Published:** 2017-02-09

**Authors:** Oren Griffiths, Nathan Holmes, R. Fred Westbrook

**Affiliations:** School of Psychology, University of New South Wales, SydneyNSW, Australia

**Keywords:** extinction, Pavlovian conditioning, animal conditioning, human learning, prediction error

## Abstract

Models of associative learning have proposed that cue-outcome learning critically depends on the degree of prediction error encountered during training. Two experiments examined the role of error-driven extinction learning in a human causal learning task. Target cues underwent extinction in the presence of additional cues, which differed in the degree to which they predicted the outcome, thereby manipulating outcome expectancy and, in the absence of any change in reinforcement, prediction error. These prediction error manipulations have each been shown to modulate extinction learning in aversive conditioning studies. While both manipulations resulted in increased prediction error during training, neither enhanced extinction in the present human learning task (one manipulation resulted in less extinction at test). The results are discussed with reference to the types of associations that are regulated by prediction error, the types of error terms involved in their regulation, and how these interact with parameters involved in training.

## Introduction

Prediction error refers to the degree of mismatch between what is expected to occur, and what actually occurs. One way to elicit a prediction error is an extinction procedure. In this procedure subjects (animals or people) are first exposed to pairings of a cue (labeled A) and an outcome (denoted +). As a consequence of having experienced several of these A+ pairings, subjects begin to respond to the cue in anticipation of the outcome. This is referred to as the acquisition phase. It is after this acquisition phase that the crucial extinction phase takes place. In the extinction phase, the cue is repeatedly presented in the absence of the outcome, referred to as A- trials. On each of these trials, the expectation of the outcome (+) elicited by the presence of the cue (A) is violated by the experimenter withholding that outcome After several errors in prediction (or prediction errors) whereby the outcome is anticipated but fails to occur, subjects learn that the cue no longer signals the outcome, and responses to the cue cease. At this point the cue-outcome association is said to be extinguished. Thus extinction learning is said to be error-driven, as it is the experience of this prediction error that drives the changes in expectation of the outcome following the cue, and thus elicits learning about that cue. The processes underlying the extinction of such responses are of theoretical and clinical significance, as extinction learning is the basis of exposure therapy, the most effective treatment for many anxiety disorders (Feske and Chambless, 1995; Taylor, 1996; Eddy et al., 2004). Therefore any procedure that purports to enhance extinction learning offers the prospect of enhancing the efficacy of its real-world applications, such as exposure therapy.

The present experiments investigated a method recently reported to enhance extinction learning (and thus exposure therapy) in adults via increasing the prediction error term on each extinction trial (Culver et al., 2015). Specifically, we tested whether two such manipulations influenced explicit extinction learning in an adult population using affectively neutral stimuli. Neutral stimuli were used in order to focus on the basic cue-outcome learning processes involved in the manipulation, and to correspondingly minimize any differential directs effects that, say, electric shock or its omission might have on learning. In order to understand these error-enhancing manipulations, it is important to first consider *how* prediction error is thought to drive learning more generally.

A range of experiments show that the formation of cue-outcome associations is regulated by prediction error. Specifically, these experiments show that the amount learned about a cue depends not only on its relation to the outcome stimulus, but also on the relation between other concomitantly present cues and that outcome. For example, the “blocking” effect demonstrated that pairings of a target cue (A) with the outcome (+), which would otherwise lead to strong learning about the relationship between the cue and the outcome, could be rendered ineffective by changing which other cues were present on that same trial. For example, if cue A was also accompanied by a second cue (B) that had been previously been trained to predict the outcome, thus rendering cue A causally redundant, then very little is learned about cue A’s relationship with the outcome; this is termed the “blocking” effect (Kamin, 1969). In prediction error terms, on the crucial compound trials (AB+ trials), the outcome (+) was already predicted by the second cue (B), and thus there was no prediction error present to drive learning about the target cue (A). Several related empirical phenomena support the role of error-correction mechanisms in acquisition learning in both animals (conditioned inhibition, Rescorla, 1969; overshadowing, Rescorla, 1970; signal validity effects, Wagner, 1969) and people (conditioned inhibition, Chapman and Robbins, 1990; blocking, Dickinson et al., 1984; super-conditioning, Aitken et al., 2000).

There is evidence from animal conditioning studies that extinction learning is also regulated by prediction error. For example, in both between- and within-subject designs, Leung et al. (2012) extinguished one target cue (A) in compound with a partner (X) that was strongly associated with the outcome, and a second target cue (B) in compound with a partner (Y) that was only weakly associated with the outcome. Thus, there was greater prediction error on AX- than on BY- trials, but the treatment of the target cues (A and B) was otherwise identical. The subsequent test of A and B revealed less conditioned responses to A, extinguished in compound with the strong associate of the outcome, X, than to B, extinguished in compound with the weak associate of the outcome, Y. The larger error across the AX- than the BY- trials increased the amount of extinction learning to A than B (see also Leung and Westbrook, 2008; Holmes and Westbrook, 2013). However, there is also evidence from animal conditioning studies that does not suggest that extinction learning depends on the size of the prediction error term. McConnell et al. (2013) used a between-group design to compare the amount of extinction learning to a target conditioned stimulus non-reinforced in compound with either two neutral cues, one neutral cue and one conditioned cue, or two conditioned cues. They found mixed evidence regarding whether extinction learning is driven by the size of the prediction error term. Consistent with the view the extinction learning is driven by prediction error magnitude, they reported that a target conditioned stimulus elicited less responding at test (more extinction) if it had been non-reinforced in compound with one neutral and one conditioned cue than in compound with two neutral cues. Yet they also reported that a target conditioned stimulus elicited less responding at test if it had been non-reinforced in compound with one neutral and one conditioned cue than in compound with two conditioned cues, suggesting that extinction learning is not just controlled by the size of the error term (see also Pearce and Wilson, 1991; Thomas and Ayres, 2004; Witnauer and Miller, 2012).

Recent studies have examined whether evidence for deepened extinction observed by Leung et al. (2012) and others (Leung and Westbrook, 2008, 2010; Holmes and Westbrook, 2013) can also be found in people. Three of these studies used an aversive conditioning procedure in which the experimenters measured both skin conductance levels and the degree to which participants expected an aversive outcome following presentation of the cue. One reason for using both measures is that skin conductance, but not expectancy, is thought to reveal implicit “non-conscious” learning (McAndrew et al., 2012) (but see Mitchell et al., 2009). The first study (Lovibond et al., 2000) examined whether extinction was greater to an excitor (a cue paired with shock) extinguished in conjunction with another excitor (prediction error was large) than to an excitor extinguished in compound with a learned safety signal (prediction error was small). However, there was no such difference on test: each of the target excitors elicited similar levels of test responding (on both measures), suggesting that the cues had failed to interact in the manner expected based on results from animal conditioning studies. In the second study, Vervliet et al. (2007) reported that extinguishing an excitor in compound with a second excitor resulted in performance at test (on both measures) comparable with pre-extinction levels of fear, suggesting that the second excitor had not only failed to enhance extinction learning about the first but had even protected the first from extinction. Again, this result suggests that the cues had failed to interact in the expected manner when presented in compound.

The third study, Culver et al. (2015), offers the most direct test of the proposal that error-correction mechanisms regulate extinction. In this between-groups study, an excitor was subjected to an initial phase of extinction, and then additional extinction either on its own or in compound with a current excitor. This was the method used by Leung et al. (2012), as it is under these conditions that many error-correction theories unambiguously predict a deepening of extinction in the compound group. Consistent with the findings reported by Leung et al. (2012), Culver et al. (2015) found that extinction in the presence of the current excitor deepened extinction of the skin conductance response: this was evidenced by greater resistance to reinstatement of such responses following exposure to the aversive event alone in the group submitted to compound extinction than in the group submitted to further extinction of the target alone. However, as in the two other aversive conditioning studies, Culver et al. (2015) failed to detect any effect of compound extinction on expectancy ratings.

Cumulatively, the literature shows that, at least under some conditions, error correction mechanisms regulate extinction of affective reactions to cues predictive of aversive events in both animals (Leung et al., 2012) and people (Culver et al., 2015). However, at present, there is no evidence that these same mechanisms regulate extinction of the explicit cue-outcome contingency in people. Whether contingency knowledge is regulated by an error-correction process remains an important question to address as cognitive factors have been shown to play a critical role in human extinction learning (for a review, see Lovibond, 2004). For example, Zeng et al. (2015; see also Raes et al., 2011) recently demonstrated that, once a cue-outcome relationship is successfully extinguished, fear of that cue can be immediately restored by providing an alternative explanation for the absence of the aversive outcome during the extinction training. That is, if people reappraise the extinction experiments as providing no evidence about the status of the underlying cue-outcome relationship (akin to using “safety behaviors” in a clinical setting; Salkovskis, 1991), then their fear of the extinguished cue is restored. This observation is consistent with the common sense notion that understanding the cause of aversive events critically influences subsequent behavioral and emotional responses (e.g., Clark, 1986). Similarly, extinction can also be rendered less effective if people aggregate across their whole experience with a cue (when it signals an aversive event in acquisition, and when it signals no such event in extinction), rather than prioritize their most recent experiences with that cue (i.e., during extinction; Collins and Shanks, 2002). Both of these phenomena, reappraisal and aggregation, indicate that effective learning will depend on how people formulate the change in the relation between cues and outcomes across extinction training.

Accordingly, the present study examined the effect of extinction on people’s knowledge of the relations between affectively neutral cues and outcomes. It specifically examined whether extinction of a target cue-outcome relationship is regulated by prediction error, which was manipulated through the associative status of cues that accompanied the target during extinction. Across both experiments, steps were undertaken to investigate the role (if any) of aggregation. Specifically, additional “filler” cues were included to assess whether people were aggregating their experiences with cues across phases when asked to assess those cues at test. Moreover, the wording of each test question was adjusted from prior experiments (e.g., Griffiths and Westbrook, 2012; Holmes et al., 2014) to indicate that people should rely on their recent experience with a cue, rather than their remote experience. However, the primary aim of Experiment 1 was to address whether extinction was directly regulated by a prediction error term, using a design analogous to those used by Lovibond et al. (2000) and Vervliet et al. (2007; see also Reberg, 1972). The target cue was extinguished in compound with a good predictor of the outcome (thus eliciting a large prediction error during extinction) while a second cue was extinguished in compound with an already-extinguished cue (thus eliciting a smaller prediction error during extinction). Experiment 2 addressed the same question with a design analogous to that used by Culver et al. (2015). The already-extinguished target cue was given further extinction in compound with another already-extinguished cue – a manipulation that has been shown to restore responding and deepen extinction learning in animal conditioning studies (Hendry, 1982; Rescorla, 2006; Leung et al., 2012). The effects of this compound extinction were assessed relative to a second cue given further extinction in isolation. If extinction learning is regulated by prediction error, the target cue in each experiment should undergo more learning than the control cue, evoking a weaker expectancy of the outcome than the control cue at test.

## Experiment 1

Both experiments used an allergist task, which is a common method for studying associative learning in people (Aitken et al., 2000; Griffiths and Mitchell, 2009). In this task, participants are asked to monitor the intake and symptoms of a fictional patient (in this case, Mrs. X) who suffers from food allergies. The foods the patient consumes are the cues, and any allergic reactions she has are the outcomes. Learning about Mrs. X’s food allergies essentially constitutes learning about cue-outcome associations in a trial-by-trial manner. Participants were additionally told that Mrs. X was undergoing chemotherapy, and that her food allergies may consequently vary across time. The design of Experiment 1 is shown in the upper row of **Table 1**. Four foods, e.g., carrots, beef, apples, pasta, labeled as cues A, B, C, and D, are of major interest. Other foods are also presented in each training phase as so-called filler cues. The manipulation of interest occurs in Phase 3 when one allergenic food (B) is extinguished in compound with a food (A) already known to be safe (AB-), whereas a second allergenic food (C) is extinguished in compound with D another allergenic food (CD-). The meals with two allergenic foods present (CD- trials) should elicit more prediction error, and drive more extinction learning for those foods (C and D), than should the meals which contain only one allergenic food (AB- trials). More precisely, the shift from A- to AB- should deepen extinction of A and protect B from extinction (Rescorla, 2006; Leung et al., 2012), while extinction of the compound containing the two allergenic foods (CD-) should be rapid and substantial. Error correction theories thus predict that these manipulations will have contrasting effects on extinction: A will protect B from extinction whereas C will facilitate extinction to D (as will D facilitate extinction of C). According to such theories, therefore, participants will judge B as less safe (or more allergenic) than C (and D) at test. We tested their knowledge of the cue-outcome associations (food-allergy associations) with forced choice items and confidence ratings for each cue.

**Table 1:** Experimental design of Experiments 1 and 2 (in the top and middle row, respectively).

	Phase 1 (8)	Phase 2 (8)	Phase 3 (8)	Forced choice
	A++	A-	AB-	B vs. D
Experiment 1	B++			A vs. C
	C++		CD-	
	D++			
	E-	E++		
	F-	F-		
	A++	A-	AB-	B vs. D

Experiment 2	B++	B-		A vs. C
	C++	C-	C-	
	D++	D-	D-	
	E-	E++		
	F-	F-		

Distracters	G-	G-	GH++	
(Both Experiment 1 and Experiment 2)	H-	H-		
	I-	I+		
	J+	J+	J+	
		K+		


*Each letter (A–K) refers to an individual food cue (e.g., carrot). The symbols (-,+,++) refer to the severity of the allergic reaction experienced by Mrs. X on each trial: “-” refers to no reaction, “+” refers to minor reaction, “++” refers to a serious reaction. The distracter items listed in the lower row were common to both experiments. The numbers in brackets in the header row refer to the number of trials per trial-type in each Phase.*

### Materials and Methods

This study was approved by the UNSW Human Research Ethics Advisory Panel, and was carried out in accordance with the recommendations of the National Health and Medical Research Committee’s National Statement on Ethical Conduct in Human Research.

#### Participants

Sixty eight second-year psychology students participated in partial fulfillment of course requirements. The mean age was 19.41 years (*SD* = 4.50), and 45 were female.

#### Design

The experiment involved three training phases followed by test. In both this and the subsequent experiment, the critical cues are labeled A–D. The remaining cues (E–K) were included to control for any relatively simple, incidental rule learning that might occur (e.g., no meal of two foods produces an allergic reaction). We did not attempt to control for more complex rules (e.g., negative patterning) for the simple reason that people view such complex cue interactions as inherently implausible (Griffiths et al., 2009).

Accordingly, our description of the training contingencies focuses on cues A–D. In Phase 1, each of these four cues (A, B, C, D) was paired with a serious allergic reaction (labeled ++). Other cues were paired with either a mild reaction (labeled +) or no allergic reaction (labeled -).

In Phase 2, one of the previously allergenic cues was extinguished (A-). In Phase 3, two compounds (AB- and CD-) were extinguished. Each of these compounds contained a cue that still predicted an allergic reaction, B and D. However, the status of its partner cue within that compound differed. B was paired with the already extinguished A, whereas D was paired with another allergenic cue, C. Therefore, the prediction error elicited by compounds AB and CD will differ, such that more prediction error will be evoked on CD- than AB- trials. Correspondingly, there will be more extinction learning on CD- than on AB- trials. The filler cues, E–K, were selected so as to balance the number of compounds that did or did not cause allergic reactions and that were followed by allergic reactions in each phase. These cues also balanced the number of cues that changed their relation to the allergenic reaction between phases, and the number of cues presented in isolation or in compound.

#### Measures

**FIGURE 1:**
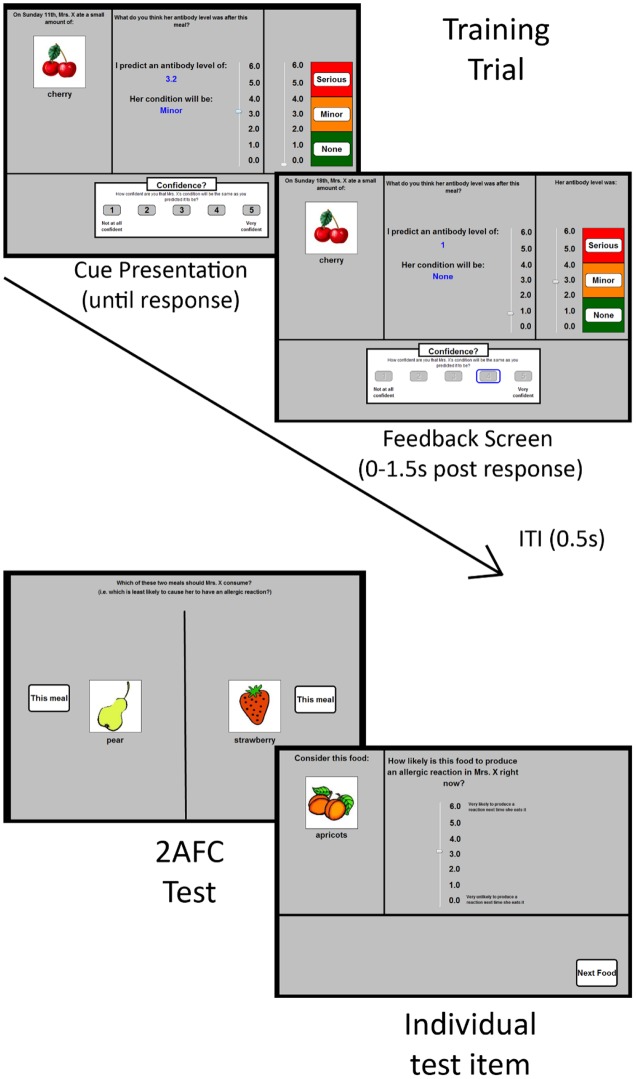
**Schematic depiction of the materials used in Experiments 1 and 2.** The **(top left)** panel shows a typical training trial in which participants were shown a food (or two foods, not shown) and were first asked to predict Mrs. X’s resultant antibody levels. They did this by manipulating the scrollbar in the panel. When that prediction was made, the participant was asked to rate their confidence using the 5-option response scale at the bottom of the screen. Feedback was then provided immediately for 1.5 s. A typical feedback screen is shown in the **(top right)** panel. It differs from the cue presentation screen in that it shows the correct value (on the right-hand scrollbar), alongside the prediction and confidence values chosen by the participant (left hand scrollbar and blue rectangle on the lower screen, respectively). Mrs. X’s antibody levels (and thus her allergic reaction response) was indicated by the right-hand response scale. The participant’s chosen confidence response remained onscreen. An intertrial interval (ITI) of 0.5 s occurred between trials, during which the preceding trial’s cue, response information, and feedback was removed from the screen. The lower two panels depict the two types of test-items. The **(lower left)** panel shows a typical forced choice test-item. The **(lower right)** item shows a typical test item in which people were asked to rate the allergenic properties of each food item individually.

There were four dependent variables: outcome predictions, confidence ratings, test ratings and forced choice responses. The first two occurred during the training phases, and the latter two occurred during the test phase. An outcome prediction was made on every training trial, following the presentation of the cue stimuli. These predictions were made using an onscreen “antibody scale” that varied between 0.0 and 6.0 in 0.1 increments (see **Figure 1**). The scale was visually divided into three categories: no reaction (0–2), minor reaction (2.1–4), and serious reaction (4.1–6). This scale was present on every trial. Participants were told that this scale indicated Mrs. X’s “antibody levels (a measure of allergic reaction severity)” after eating each food (see Griffiths et al., 2011). Each time they moved the scrollbar to make a prediction, the numeric value of the scrollbar (e.g., 1.6) was shown on-screen, as was the category of reaction (none, minor, serious) that corresponded to that prediction.

The confidence scale consisted of a five-point scale (where 1 was “not at all confident” and 5 was “very confident”), whereby people rated their confidence in each outcome prediction. The scale was shown in the lower portion of the screen following each outcome prediction rating (see **Figure 1**).

The test ratings were made individually for each food cue on separate screens. At the initiation of this test phase, participants were told that Mrs. X was undergoing medical treatment and therefore had to keep her antibody levels within the normal range. Consequently, the participant had to identify which foods were or were not safe for Mrs. X to eat right now by rating each food on a 0.0–6.0 scale (where 0 was “very unlikely to produce a reaction next time she eats it” and 6 was “very likely to produce a reaction next time she eats it”). Like the scale anchors, the wording of the test question on the screen (“how likely is this food to produce an allergic reaction in Mrs. X right now?”) and also the wording of the instructions for this test phase both emphasized the importance of the participants rating the *current* status of the food, rather than providing a rating based on averaging over the history of their experience with that food (see Collins and Shanks, 2002).

On each forced choice test item, participants were shown two meals (which each consisted of a single food), and they were asked to click on the meal that would be safer for Mrs. X to eat at that moment. The left/right positions of each food cue was randomly determined for each participant.

#### Procedure

The experiment was conducted in classes of approximately 20 students per class. The task was computer-based. Participants were first instructed to assume the role of an allergist who had to learn which foods made a new patient (Mrs. X) feel ill and those which were safe for her to consume.

On each trial, participants were shown a meal containing either a single food (e.g., the word “carrots” and a color line drawing of carrots) or two foods (e.g., “beans and broccoli” and a line drawing of each). They were asked to predict whether consumption of the meal would cause an allergenic reaction (see **Figure 1**) using the outcome prediction scale (see *Measures*). Foods were randomly assigned to cue-types (e.g., A, B, C…K) for each participant. There was no time limit to make a prediction. Once a prediction had been made, participants indicated their confidence using the confidence scale (see Measures).

The scrollbar was then inactivated, and corrective feedback was provided onscreen for 1.5 s. Specifically, participants were shown Mrs. X’s actual antibody level alongside their own estimate on the visual analog scale (they were not given a numeric value as feedback). The position of the feedback indicator on the visual analog scale was jittered around the middle values of each category. This meant that the value given as feedback was not identical on each +, - or ++ trial: antibody levels were randomly selected from a uniform distribution between 0.4 and 1.3 on each – trial, 2.5 and 3.4 on + trials, and between 4.6 and 5.5 on ++ trials. This meant there was always some degree of uncertainty (and therefore potentially prediction error to drive learning) on each trial.

The order of the trials in each of the three phases was randomized with the constraint that all trial types were shown once before any trial type was shown a second time. There were eight instances of each trial type in each phase, yielding 176 trials in total, and the interval between trials was 0.5 s. The transition between phases was not signaled.

Upon completing phase 3, participants were tested. Participants first completed two forced choice test items (see Measures), between cues A and C, and between cues B and D. The order of presentation of these items was randomized for each individual. They then completed test ratings for each cue A–K (see Measures). The cues were presented individually, and the order was randomized for each individual.

### Results

#### Exclusion Criterion

We first examined whether participants learned the initial training contingencies shown in Phase 1. Outcome predictions on the 0.0 to 6.0 scale were coded in 0.1 increments to yield a score of 0–60 for each trial or test item. Participants’ mean outcome predictions for cues A–D in the last half (four trials) of Phase 1 training were averaged, yielding a value between 0 and 60. All of these trials were consistently paired with a serious allergic reaction in Phase 1 (4.0 or above). Any participant with a mean rating for these cues of less than the midpoint of the response scale (i.e., less than 30 out of 60) was excluded. This resulted in the removal of nine participants (13%). The remaining analyses were performed on the data from the remaining 59 participants. It is worth noting that the removal of these participants from the statistical analysis did not change the pattern of means in any inferential test in either Experiment 1 or 2. Instead their removal reduced variance (likely noise) from the data. All inferential statistics controlled the two-tailed Type I error rate at 5%, and confidence intervals were constructed at the same confidence level.

#### Outcome Prediction Accuracy and Confidence Ratings

**FIGURE 2:**
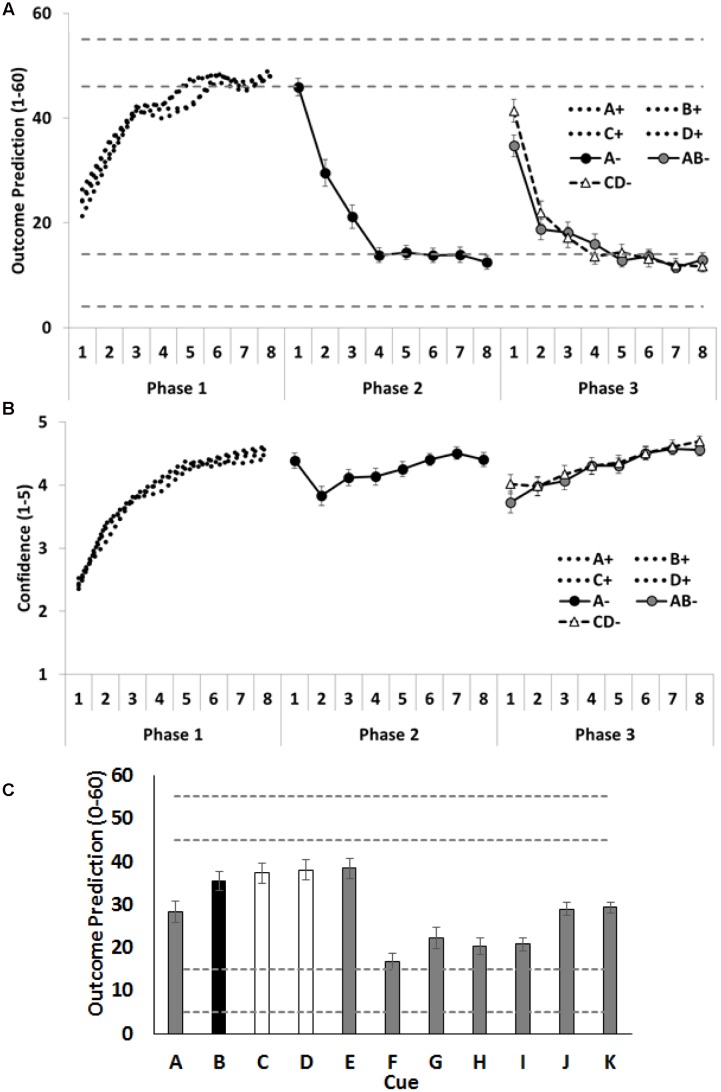
**Performance in Experiment 1.** **(A)** Depicts outcome prediction responses across the three training phases for the critical cues A–D. The broken horizontal lines on **(A,C)** indicate the range of outcome values (allergic response severity, 0–60; corresponds to 10 times the 0.0–6.0 value seen in **Figure 1**) that could occur on a ++ trial (the upper range) or on a – trial (the lower range). **(B)** Depicts confidence ratings for these same trial-types across the three training phases. **(C)** Shows people’s test ratings for the individual cue test items. In panel C the critical cue (B) is shown as a black column, and the comparison control cues (C and D) are shown as white columns. Error bars indicate standard error of the mean in all panels.

Outcome predictions and confidence ratings for the critical cues (A–D) across all three training phases are shown in **Figures 2A,B**. Inspection of the figures indicates that participants rapidly learned the contingencies across each training phase. This was evident in their increasing accuracy and confidence across each training phase. Notably, confidence dropped on the second trial of Phase 2, after participants experienced direct disconfirmation of their prior expectations regarding cue A on the initial trial of Phase 2. However, the question of primary theoretical interest in these data is their initial responses to the AB and CD compounds in Phase 3. We hypothesized that people would anticipate the outcome less strongly on the initial AB trial (with one allergenic cue and one extinguished cue) than on the initial CD (with two allergenic cues) trial. This result would be indexed by lower outcome predictions and lower confidence for AB than for CD on the first trial of Phase 3. The initial outcome predictions for compound CD did in fact significantly exceed that for compound AB, *F*(1,58) = 5.61, *p* = 0.02, $\eta_{p}^{2}$ = 0.09, CI [1.04, 12.35], but no difference was found between AB and CD on the confidence ratings, *F*(1,58) = 3.22, *p* = 0.08, $\eta_{p}^{2}$ = 0.05, CI [-0.03, 0.61].

#### Test Ratings and Forced Choice Responses

Mean causal ratings for cues A–K are shown in **Figure 2C**. Two orthogonal contrasts were used to examine the amount of extinction for the critical cues A–D. The first contrast compared test ratings for cue B (extinguished in compound with the already extinguished A), with the average of cues C and D (both of which were allergenic when combined into the CD- compound). No significant differences were observed, *F* < 1, *p* = 0.41, $\eta_{p}^{2}$ = 0.01, CI [-14.71, 6.13]. Given this absence of a significant difference, the power analysis (Faul et al., 2007, 2009) showed there was sufficient power (*1 –*β = 0.8) to detect a small to medium effect size (*f* = 0.24). The implied population effect size for the contrast testing B vs the average of C and D was very small (*f* = 0.11; $\eta_{p}^{2}$ = 0.01) and would have required 281 people to find any effect of this magnitude (with *1 –*β = 0.8). The second contrast examined whether the additional extinction training given to cue A resulted in more extinction for that cue, than to the less frequently extinguished cues B, C and D; it did, *F*(1,58) = 9.18, *p* = 0.004, $\eta_{p}^{2}$ = 0.14, CI [8.95, 43.77].

There were two additional contrasts. The first examined whether participants used the most recent status of each cue or had aggregated over their prior experiences with that cue to generate choice on test. To assess these alternatives, we compared the test ratings given to cue J, which was paired with a minor reaction across all 3 phases, against the average of cues G and H, which were paired with no reaction in Phases 1 and 2, but were paired with severe reaction in the final phase. If people were aggregating their experience across all three phases, they should rate J higher than the recently reinforced G and H; in fact, this was the case, *F*(1,58) = 14.47, *p* < 0.001, $\eta_{p}^{2}$ = 0.20, CI [7.35, 23.67]. The second contrast compared two cues, I and K, each of which had been associated with minor reaction, when last shown. However, the prior training was that I had been associated with no reaction, whereas K had no prior training. If participants were influenced by the history of a cue prior to its most recent presentation, they should rate K higher than I; in fact, this was the case, *F*(1,58) = 25.20, *p* < 0.001, $\eta_{p}^{2}$ = 0.30, CI [5.09, 11.85]. Taken together, therefore, these results show that participants were influenced by the history of the cue in judging its effectiveness on test, although this does not preclude them from also using the most recent status of a cue in these judgments.

The forced choice data showed a similar pattern. When required to choose between B and D, 32 (54%) participants chose B as the safer food. A binomial test revealed that this did not significantly differ from chance, *p* = 0.60, CI [24.31, 39.69]. By contrast, significantly more participants chose A (68%) as safer than C, *p* = 0.009, CI [23.31, 47.69], indicating that the additional extinction training for A resulted in additional learning for this cue. It is possible that the overall lack of a difference between B and D was obscured by a number of people (at least 32% of the sample) who did not learn that A (extinguished in both Phases 2 and 3) was safer than C (just extinguished in Phase 3). If these participants effectively treated A and C as equivalently extinguished, then there would be no reason to expect a difference in the amounts learned about their partner cues, B and D, respectively. Therefore, we conducted a second analysis of the B versus D forced choice data on only those participants who chose A as safer than C. Of the 44 participants who chose A as safer than C, 28 chose B, the partner of A, as safer than D, the partner of C. This difference was not statistically significant, *p* = 0.16, 95% CI [18.60, 31.40], confirming that the pattern of responding to the target cues, B and D, did not vary with differences in responding to their within-compound partners, A and C.

### Discussion

The compound of two allergenic cues (CD-) elicited higher outcome predictions at the beginning of Phase 3, indicating that people initially expected the outcome more on these trials than on the initial AB- trials of Phase 3. This demonstrates that the manipulation was effective and that prediction error was greater across the CD- than the AB- trials; hence, more associative change should have accrued to C and D than to B. However, on the subsequent test, participants did not rate C and D as less allergenic than B nor did they choose D as safer than B. In fact, the direction of the means was in the opposite direction (B > D), both when considering all participants and just those individuals who demonstrated knowledge of A’s additional extinction training (by choosing A as safer than C on test). This pattern of results is broadly consistent with previous examinations of compound extinction in human causal learning tasks (Griffiths and Westbrook, 2012; Holmes et al., 2014). It is also consistent with the results from the two aversive conditioning with humans (Lovibond et al., 2000; Vervliet et al., 2007) that also failed to detect any facilitatory effect of extinguishing a compound composed of two aversively conditioned stimuli, as measured by skin conductance and expectancy ratings. This absence of a difference between the target cues (B and D) may be due, in part, to people aggregating over their entire experience with these cues, rather than prioritizing their recent experience (despite the explicit onscreen instructions to do so). Discussion of this issue is withheld to the Section “*General Discussion*.”

## Experiment 2

In contrast to the results reported by Lovibond et al. (2000), Vervliet et al. (2007), and Culver et al. (2015) found enhanced extinction for cues trained in compound over cues trained in isolation (on skin conductance and responsiveness to a reinstating outcome, but not on outcome expectancy measures). Culver et al argued that their results were due to having subjected each of the critical cues to extinction before the compound extinction.

Accordingly, our second experiment used a design analogous to that of Culver et al to provide a further examination of the role played by prediction error in extinction of a cue-outcome contingency. This design again involved manipulating prediction error during extinction learning by presenting some cues in compound and others in isolation (see Hendry, 1982). However, in this experiment, the manipulation occured *after* all of the target cues (A-, B-, C-, and D-) had been individually extinguished. Two of those cues (A and B) were then given further extinction in compound (i.e., AB- trials). The rational was that A and B have each retained some association with the outcome, but one that is not sufficient to drive responding on its own. By presenting these two individually ineffectual cues together, their combined capacity to predict the outcome should cross the threshold to elicit renewed prediction of the outcome (Rescorla, 2006). Because these AB- trials therefore elicit some degree of prediction error, this error will drive further extinction learning for these cues. This was tested by comparing the cues given additional compound extinction (A and B), with two control cues (C and D) given the same amount of extinction training but in an individual format (i.e., on separate C- and D- trials). If extinction of causal judgements is regulated by prediction error, extinguished cues that receive additional extinction in compound (A and B) should be treated as safer at test than extinguished cues that received additional extinction in isolation (C and D). As far as we are aware, this hypothesis has not yet been investigated in a human causal learning task.

### Materials and Methods

#### Participants

Seventy six second-year psychology students participated in partial fulfillment of course requirements. The mean age was 20.30 years (*SD* = 3.65), and 60 were female.

#### Design

The design of the experiment is summarized in the second row of **Table 1**. The four cues (A, B, C, and D) of major interest were each paired with a serious allergic reaction (antibody scores > 4.0) in Phase 1. Then in Phase 2, each of the four cues (A–D) no longer produced that allergic reaction, and were instead followed by no allergic reaction (i.e., normal antibody scores, <20). In the final training phase, Phase 3, A and B were shown together and produced no allergic reaction (AB- trials). The other critical cues, C and D, were each shown individually, and continued to produce no allergic reaction (C- and D- trials).

#### Measures

The same measures were used as were used in Experiment 1.

#### Procedure

The procedure was identical to Experiment 1, and only differed with regards to the training contingencies detailed in **Table 1**.

### Results

#### Exclusion Criterion

The same exclusion criterion as used in Experiment 1 was applied to the present data set. It resulted in the removal of data from three participants (4%).

#### Outcome Prediction Accuracy and Confidence Ratings

**FIGURE 3:**
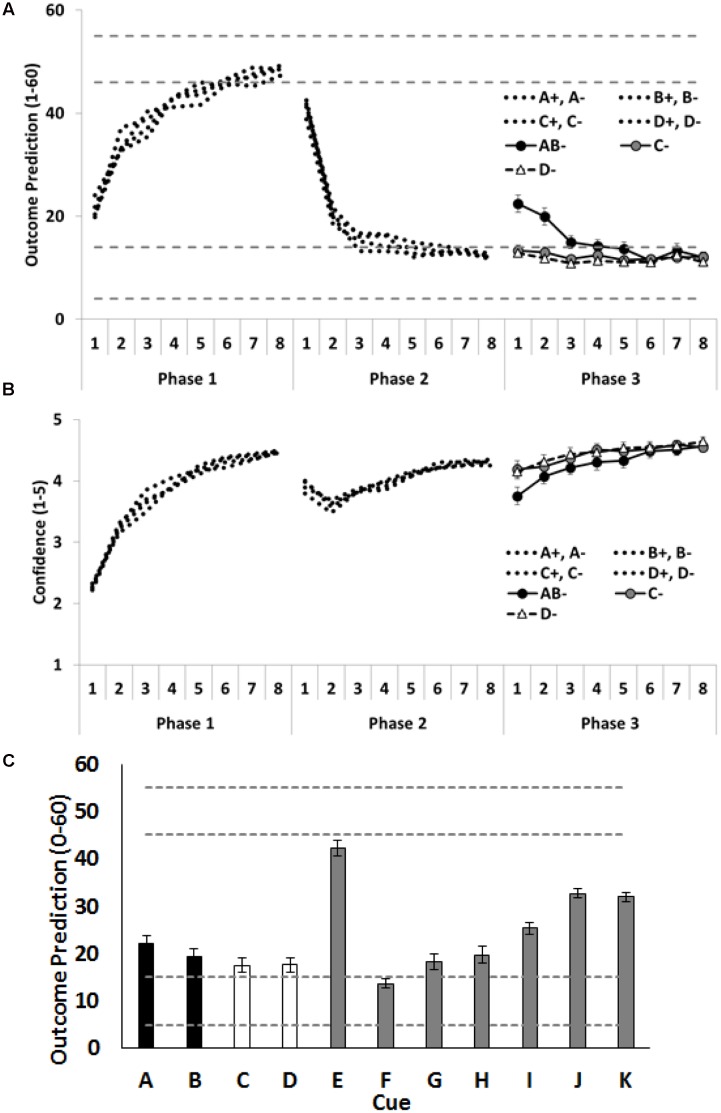
**Performance in Experiment 2.** **(A)** Depicts outcome prediction responses across the three training phases for the critical cues A–D. The broken horizontal lines on **(A,C)** indicate the range of outcome values (allergic response severity, 0–60; corresponds to 10 times the 0.0–6.0 value seen in **Figure 1**) that could occur on a ++ trial (the upper range) or on a – trial (the lower range). **(B)** Depicts confidence ratings for these same trial-types across the three training phases. **(C)** People’s test ratings for the individual cue test items. The critical cues (A and B) are shown as black columns, and the comparison control cues (C and D) are shown as white columns. Error bars indicate standard error of the mean in all panels.

As shown in **Figures 3A,B**, Participants rapidly learned the training contingencies: outcome predictions increased (Phase 1) and then decreased (Phases 2 and 3); and confidence in predictions increased across each training phase. Again, our primary theoretical interest concerns how participants treat the critical cues A, B, C and D at the beginning of Phase 3. As predicted, combining the extinguished A and the extinguished B into a compound restored responding, as indicated by the higher outcome predictions for compound AB than for the individually presented C and D. To test this, the average of the outcome predictions on C- and D- trials was compared with the outcome predictions given on AB- trials. Again, the first trial is the data point of most interest, as this is the time at which the outcome predictions based on the compound can be assessed prior to corrective feedback for these predictions. On the first trial, participants gave higher outcome predictions for the AB compound than the average of C- and D- trials, *F*(1,72) = 28.78, *p* < 0.001, $\eta_{p}^{2}$ = 0.29, CI [11.74, 25.63]. Moreover, they were less confident about their prediction on the initial AB- trial than on the initial C- and D- trials, *F*(1,72) = 10.99, *p* = 0.001, $\eta_{p}^{2}$ = 0.13, CI [0.33, 1.33]. As can be seen in **Figure 3**, this difference between the AB- trials and the C-/D- trials did not persist. By the end of training people were making the same predictions on both the compound (AB-) and the individual (C- and D-) trials with the same levels of confidence.

#### Test Ratings and Forced Choice Responses

**Figure 3C** shows the mean causal ratings for cues A–K. A single contrast compared test ratings for the average of the cues, A and B, that had received additional extinction in compound, with the average of the cues, C and D, that had each received additional extinction in isolation. The contrast showed that C and D received significantly lower test ratings, *F*(1,72) = 4.58, *p* = 0.04, $\eta_{p}^{2}$ = 0.06, CI [0.42, 11.99] than A and B, indicating that participants learned more about the cues that had been subjected to additional extinction in isolation than in compound. This is the opposite finding to that reported by Leung et al. (2012) using a fear response in rats and Culver et al. (2015) using a skin conductance measure in people.

As in the previous experiment, participants appeared to base their judgements on the aggregated rather than the most recent value of a cue. Specifically, participants rated J, paired throughout with a minor reaction, as more allergenic than G and H, each paired with a severe reaction but only in the final phase, *F*(1,72) = 58.59, *p* < 0.001, $\eta_{p}^{2}$ = 0.24, CI [20.17, 34.37]. Participants also rated I, initially paired with no reaction and then with a reaction in Phase 2, as less allergenic than K, paired with a reaction just in Phase 2, *F*(1,72) = 23.20, *p* < 0.001, $\eta_{p}^{2}$ = 0.45, CI [3.87, 9.36].

The forced choice data showed that 35 people chose C as safer than A (52%), and 43 (59%) chose D as safer than B. Because A and B were treated identically, as were cues C and D, inferential statistics were conducted on the choices of A + B versus the choices of C + D. There were more choices of C + D (55%) than of A + B, but this difference was not statistically significant, *p* = 0.22, CI [69.05, 92.94]. We also examined only those participants who chose both A and B (19 people) as compared with those who chose both C and D (27 people; 59%). This difference was also not significant, *p* = 0.30, CI [20.17, 33.83].

### Discussion

Compounding two previously extinguished cues (A- and B-) transiently restored outcome predictions. These predictions were significantly higher on the initial AB- trial of Phase 3 than for on the initial C- and D- trials. This means that prediction error across the additional AB- trials should have also been greater than across the additional C- and D- trials and, hence extinction learning about A and B should have been enhanced relative to that learning about C and D. However, this did not occur: in fact, the test measure of outcome expectancy revealed that the individually extinguished C and D were rated as significantly safer (less allergenic) than the otherwise matched, but compound extinguished, A and B. The forced choice items were in the same direction as their outcome expectancy ratings, but no significant differences were observed. In sum, the compound manipulation used to restore responding was successful but the deepening of extinction learning across additional extinction of that compound was not confirmed: if anything, that additional extinction of the compound appeared to impair extinction learning in this human learning analog.

## General Discussion

Two experiments examined whether extinction of cue-outcome contingency knowledge is regulated by an error-correction process: specifically, whether manipulations that maintain or restore outcome expectancies in extinction can facilitate or deepen the learning that occurs when a cue is presented in the absence of its expected outcome. This deepening has been observed in extinction of conditioned fear in rats (Leung et al., 2012) and extinction of skin conductance responses in people (Culver et al., 2015). This effect is predicted by theories of associative learning (e.g., Rescorla and Wagner, 1972; Wagner, 1981) which hold that all the cues present on a trial are used to calculate the error whose size determines the amount of associative change and whose sign (positive or negative) determines the nature of the change (excitatory in the case of acquisition or inhibitory in the case of extinction).

In each of the two experiments, we used a different manipulation to maintain or restore outcome expectancies across extinction of the cue-outcome contingencies. In Experiment 1, a target cue, D, was extinguished in compound with a non-extinguished cue, C, and the consequences for its extinction were assessed relative to a control cue, B, extinguished in compound with an already-extinguished cue, A. Critically, this manipulation was effective in generating differences in responding such that the CD compound was treated as more allergenic than the AB compound, which should have served to increase the size of the prediction error on CD- trials relative to AB- trials. However, the levels of test responding to B and D revealed no evidence that the larger error on CD- trials had deepened the extinction of D relative to that of its control cue, B: both cues were rated as equally allergenic, and when forced to make a choice, equal numbers of people chose B as more allergenic than D, and D as more allergenic than B. Thus, just as Lovibond et al. (2000) and Vervliet et al. (2007) failed to find any evidence for facilitated extinction of skin conductance responses or expectancy ratings to a cue predictive of shock, Experiment 1 failed to find any evidence for facilitated extinction of cue-outcome contingency knowledge in a causal judgment task.

These results clearly offer no support for the hypothesis that extinction of cue-outcome contingency knowledge is regulated by prediction error. However, they should not be taken as evidence *against* that hypothesis. The design used in Experiment 1, which is based on that used by Lovibond et al. (2000) and Vervliet et al. (2007), is one for which the predictions of error-correction theories are parameter dependent. Specifically, as the target cue, D, was only ever extinguished in compound with a non-extinguished partner, C, error-correction theories predict that its extinction should have been facilitated (i.e., participants should have abandoned responding to D at a faster rate than they abandoned responding to B), but, critically, that extinction of D would not necessarily have been deepened: that is, such theories hold that with sufficient extinction the net strengths of D and B at the end of extinction will in fact be equal. Hence, rather than showing that extinction of cue-outcome contingency knowledge is *not* regulated by prediction error, an alternative explanation for the results of Experiment 1 is that B and D had been extinguished to their common low asymptote, and hence, there was no opportunity for detecting any facilitation of extinction to D relative to B. However, it is noteworthy that most other cues were given lower ratings at test than either B or D (see **Figure 1**), which diminishes the conclusion that these cues were both at their lowest, asymptotic value.

In any case, there is no ambiguity in error-correction theories’ predictions of deepened extinction in Experiment 2; a deepening that has been found with affective reactions in aversive conditioning procedures with rats (Leung et al., 2012) and people (Culver et al., 2015). In this experiment, four allergenic cues, A, B, C, and D, were each presented alone during an initial phase of extinction. The target cue, B, then received additional extinction in compound with one of the other extinguished cues, A, while control cues C and D continued to be extinguished alone. Critically, the compounding of two already-extinguished cues, AB, restored the expectation of the outcome relative to continued presentations of C and D alone: that is, the AB compound was treated as more allergenic than presentations of either C or D alone, and, hence, the size of the error on the AB- trials should have been greater than on C- and D- trials. However, here again, ratings of the individual cues at test revealed no evidence that the larger error on AB- trials had deepened the extinction of A and B relative to that of the control cues, C and D. In fact, if anything, we observed the opposite result: A and B were rated as more allergenic than C and D. Thus, unlike the findings reported by Leung et al. (2012) and Culver et al. (2015), the present experiment failed to find any evidence for deepened extinction of (affectively neutral) cue-outcome contingency knowledge in a causal judgment task.

One way of reconciling the findings reported by Leung et al. (2012) and Culver et al. (2015) with those reported in the present study is to assume that there are differences across the protocols (aversive conditioning versus causal judgments) in the extent to which the effects of compound extinction generalize to testing (e.g., Pearce, 1987). Specifically, there was less generalization of compound extinction in the present study than in the two previous ones, possibly as a function of differences in cue duration and trial rate, and/or the types of association formed in extinction (affective versus contingency knowledge). For example, in the Leung et al. (2012) and Culver et al. (2015) studies, the cues were of fixed duration (30 and 8 s, respectively) and the interval between trials in acquisition and extinction was relatively long (120 s and ∼25 s, respectively); whereas in the present study (and other studies of human causal judgments), cues were presented on screen for as long as it took participants to respond (typically, 1–2 s), and the interval between the response and the subsequent trial was much shorter (0.5 s). It has previously been shown that both of these parameters can influence the likelihood of inhibitory or excitatory learning in procedures where both types of learning are possible (i.e., second-order conditioning; Karazinov and Boakes, 2007); perhaps this may also influence the propensity to generalize from configural to elemental representations.

Another way of expressing the same point is that the methods of testing used here were not sufficiently sensitive to detect the effects of compound extinction reported previously. Indeed, the self-rated test items in this task have an inherent limitation with respect to the information participants are likely to use when answering them. Collins and Shanks (2002) noted that when people are asked to rate the likelihood that an outcome will follow a cue, their answer critically depends on when they are asked. If asked during the training phase, people are more influenced by their recent experience with the cue and the outcome, whereas if asked at the end of training (in a test phase), people are more likely to aggregate across all of their experiences with the cue and outcome. Such aggregations would minimize any differences in recent extinction training, such as those investigated here. To minimize the likelihood of people responding at test based on averaging, we emphasized to participants that they should rely upon their recent experience (how would Mrs. X react *now* if she ate this food). This was achieved by adjusting the cover story of the allergist task, and altering the wording of the test question and response items. First, people were told from the outset that Mrs. X would soon undertake a medical procedure during which time she could not afford to have an allergic reaction. Therefore, people were asked to review her recent meal intake and allergic responses (the training phase), before acting as allergists to advise which foods were most likely to be safe for her during the procedure (the test phase). Each trial presented an incrementally increasing date on the screen, and the date of the test phase items followed immediately those of the training phase. Second, the wording of the instructions for the test phase again emphasized people needed to indicate which foods were safe for Mrs. X “right now.” The test question shown on each test item asked “How likely is this food to produce an allergic reaction in Mrs. X right now?” and the anchors on the response scrollbar similarly included the word now.

Despite these efforts, the analyses of the distractor cues (G, H, I, J, K) in both experiments suggest that the test ratings were influenced by their experience with each cue prior to the final phase in which that cue was shown. For instance, at the time of test, cues G and H were paired most recently with a strong allergic reaction (outcome ++) where cues J and K were most recently paired with a mild allergic reaction (outcome +). Yet across both experiments, cue J was rated higher than G and H at test. Such data suggest that our prominent, repeated verbal instructions were not, or not completely, successful in directing participants to base their test ratings just on their recent experience with a cue rather than on their history of experience with that cue.

## Conclusion

The present study showed that extinction of a target cue in compound with either a second allergenic cue (Experiment 1) or a second extinguished cue (Experiment 2) led to a maintenance or restoration of outcome expectancies across compound extinction. However, even though both manipulations increased the prediction error during the critical phase of compound extinction, neither facilitated nor deepened extinction learning of cue-outcome contingency knowledge. These results are similar to those reported by Holmes et al. (2014), and on the face of it, stand in contrast to findings reported by Leung et al. (2012) and Culver et al. (2015) showing that extinction of affective reactions to a target cue can be deepened. It is possible that this difference in conclusion relies upon the parameters of the acquisition, extinction and test procedures used, and also upon people’s propensity to use all of their prior experience with a cue, rather than only their most recent experiences. If so the efficacy of enhancing exposure therapy using these methods may depend critically on the specific spacing, duration and format of both the exposure sessions and any anxiety-relevant events that have occurred in the past. Because a number of these properties are typically outside of the therapists’ control, it remains unclear whether the present prediction-error enhancing methods will readily generalize to clinical practice. These questions remain for future research.

## Author Contributions

OG contributed to: the construction of materials, the design of the experiment, the statistical analysis, the interpretation of the data, the preparation of the manuscript. NH contributed to: the statistical analysis, the interpretation of the data, the preparation of the manuscript. RW contributed to: the design of the experiment, the interpretation of the data, the preparation of the manuscript.

## Conflict of Interest Statement

The authors declare that the research was conducted in the absence of any commercial or financial relationships that could be construed as a potential conflict of interest.
